# An End-to-End Multi-Channel Convolutional Bi-LSTM Network for Automatic Sleep Stage Detection

**DOI:** 10.3390/s23104950

**Published:** 2023-05-21

**Authors:** Tabassum Islam Toma, Sunwoong Choi

**Affiliations:** School of Electrical Engineering, Kookmin University, Seoul 02707, Republic of Korea; tabassum2485@kookmin.ac.kr

**Keywords:** automatic sleep stage detection, convolutional neural network (CNN), bidirectional long short-term memory (Bi-LSTM), electroencephalogram (EEG), electrooculogram (EOG)

## Abstract

Sleep stage detection from polysomnography (PSG) recordings is a widely used method of monitoring sleep quality. Despite significant progress in the development of machine-learning (ML)-based and deep-learning (DL)-based automatic sleep stage detection schemes focusing on single-channel PSG data, such as single-channel electroencephalogram (EEG), electrooculogram (EOG), and electromyogram (EMG), developing a standard model is still an active subject of research. Often, the use of a single source of information suffers from data inefficiency and data-skewed problems. Instead, a multi-channel input-based classifier can mitigate the aforementioned challenges and achieve better performance. However, it requires extensive computational resources to train the model, and, hence, a tradeoff between performance and computational resources cannot be ignored. In this article, we aim to introduce a multi-channel, more specifically a four-channel, convolutional bidirectional long short-term memory (Bi-LSTM) network that can effectively exploit spatiotemporal features of data collected from multiple channels of the PSG recording (e.g., EEG Fpz-Cz, EEG Pz-Oz, EOG, and EMG) for automatic sleep stage detection. First, a dual-channel convolutional Bi-LSTM network module has been designed and pre-trained utilizing data from every two distinct channels of the PSG recording. Subsequently, we have leveraged the concept of transfer learning circuitously and have fused two dual-channel convolutional Bi-LSTM network modules to detect sleep stages. In the dual-channel convolutional Bi-LSTM module, a two-layer convolutional neural network has been utilized to extract spatial features from two channels of the PSG recordings. These extracted spatial features are subsequently coupled and given as input at every level of the Bi-LSTM network to extract and learn rich temporal correlated features. Both Sleep EDF-20 and Sleep EDF-78 (expanded version of Sleep EDF-20) datasets are used in this study to evaluate the result. The model that includes an EEG Fpz-Cz + EOG module and an EEG Fpz-Cz + EMG module can classify sleep stage with the highest value of accuracy (*ACC*), Kappa (*Kp*), and *F1 score* (e.g., 91.44%, 0.89, and 88.69%, respectively) on the Sleep EDF-20 dataset. On the other hand, the model consisting of an EEG Fpz-Cz + EMG module and an EEG Pz-Oz + EOG module shows the best performance (e.g., the value of *ACC*, *Kp*, and *F1 score* are 90.21%, 0.86, and 87.02%, respectively) compared to other combinations for the Sleep EDF-78 dataset. In addition, a comparative study with respect to other existing literature has been provided and discussed in order to exhibit the efficacy of our proposed model.

## 1. Introduction

Sleep quality assessment via sleep staging is very essential for the human body in order to maintain good health and well-being as any irregularities in the sleep cycle may render pernicious consequences to physical and mental health, including acute sleep apnea, exhaustion, a lack of focus, or metabolic issues such as diabetes and obesity [1,2]. In the long run, these can increase the chance of developing a variety of other illnesses. Therefore, several methods conducted by a human specialist were developed based on overnight polysomnography (PSG) signals that electroencephalogram (EEG), an electrooculogram (EOG), and an electromyogram (EMG), which have been in use for a long time. Most recently, these traditional manually operated methods have been replaced by automatic detection methods following the guidelines of the American Academy of Sleep Medicine (AASM) or Rechtschaffen and Kales (R&K) as the manual sleep stage scoring is tedious, time-consuming, expensive, and sensitive to human errors [3,4].

In recent years, the overwhelming progress in the fields of machine learning (ML) and deep learning (DL) has accelerated its expansion in diverse application domains ranging from computer vision to natural language processing followed by predictive analysis, time-series forecasting, and digital healthcare [5,6,7,8]. Motivated by these unprecedented successes in different domains, ML/DL-based schemes have received considerable attention from the sleep research community. More specifically, DL-based methods, which include convolutional neural networks (CNN), recurrent neural networks (RNN), and a variant of RNNs called long short-term memory (LSTM) network, have been extensively explored to design automatic sleep scoring because of their powerful capabilities of capturing spatial and temporal features from the complex data distribution and mapping them in decision making with higher accuracy without manual intervention [9,10,11,12,13,14,15,16,17,18,19,20,21,22,23,24,25,26,27]. In addition, the ever-growing annotated sleep databases and their availability has also stimulated sleep researchers to design and develop novel sleep scoring methods and to test under powerful DL frameworks. 

Despite comprehensive studies on DL-based automatic sleep staging modeling, none of them have depicted gold standard performance (e.g., reliable and robust results) because of several drawbacks that include the use of single-channel data, the absence of of potential features, data variability, and data inefficiency [13,28,29]. In order to improve performance, researchers have adopted data fusion or feature fusion techniques from multiple data channels, mostly from two distinct channels of the PSG recording. Although a significant improvement has been achieved, designing multi-channel DL-based architecture requires a large amount of computational resources leading to a high resource cost and an increased training time of the model. In addition, the choice of two distinct channel’s data randomly can lead to poor performance. In this article, we aim to develop a multi-channel convolutional Bi-LSTM network model that will take input data from multiple channels of the PSG recording (e.g., EEG Fpz-Cz, EEG Pz-Oz, EOG, and EMG) and exploit the extracted multi-channel spatiotemporal data features for automatic sleep staging. We have employed the transfer-learning concept circuitously to reduce the burden of a high computational cost and reduce overall training time. First, a dual channel two-layer CNN-incorporated Bi-LSTM network module has been designed to learn the extraction of spatiotemporal features from the data of any two distinct channels of the PSG recording. Instead of LSTM layers, Bi-LSTM layers are utilized in the proposed model because Bi-LSTM layers are capable of addition training, which utilizes input data twice for training (i.e., first from left to right and then from right to left), and, hence, are prone to achieve better performance [30]. However, two such modules are concatenated later and trained following the principle of transfer learning to learn the extraction of features from four channels’ data and to fuse those features to make predictions with higher accuracy. The main contributions of this paper are summarized as follows:

▪ This paper proposes a multi-channel, more specifically a four-channel, convolutional Bi-LSTM network for automatic sleep scoring with high accuracy.
▪ In the proposed model, a dual-channel two-layer CNN-incorporated Bi-LSTM network module is designed and pre-trained utilizing data from any two distinct channel signals of the PSG recording.
▪ Once the pre-training is finished and the dual channel module is validated, using two such pre-trained modules, a four-channel model has been developed, and the concept of transfer learning is employed circuitously to reduce the burden of a high computational cost and reduce the overall training time.
▪ In the dual-channel module, convolutional layers are employed to extract spatial features from two channel PSG recordings, and these extracted spatial features are coupled with the input at every level of the BI-LSTM network to extract and learn rich temporally correlated features.
▪ Finally, we have evaluated the performances of the proposed model on the Sleep EDF-20 and Sleep EDF-78 datasets. In addition, we compared the performance with other existing works.

## 2. Related Works

In recent years, different types of DL-based schemes have been developed in sleep staging to avoid the complexity associated with the establishment of mathematical models and the extraction of handcrafted features. Cui et al. proposed a CNN-based automatic sleep stage classification method that takes 30 s fine-grained segments of multi-channel EEG signals in multiscale entropy analysis as a time-series input [9]. In order to construct input time series based on the fine-grained segments, the authors of [9] reorganized the posterior and current segments, and the size of the time series was decided based on the scale chosen depending on the fine-grained segments. A 14-layer CNN classifier was developed in [10] for automatic sleep scoring based on single-channel EEG. The model also takes as input the 30 s epoch to be classified along with two preceding epochs and one following epoch for temporal context. A Deep Learning (DL) model named DeepSleepNet was developed for the automatic classification of sleep stages from single-channel, raw electroencephalogram (EEG) data [11]. DeepSleepNet uses CNNs to extract time-invariant features and bidirectional long short-term memory to learn transition rules among sleep stages directly from EEG epochs. This approach effectively captures temporal information and enables the identification of the next sleep stage. One study [12] suggested a framework for autonomous sleep staging based on CNNs and then offered a straightforward yet effective CNN architecture to power the system. The authors of the paper stated that their combined approach for classification and prediction has the ability to overcome the limitations of the traditional classification method while also enhancing the accuracy of the model. In [13], the authors integrated deep learning with complex networks to present a novel temporal-graph-fused dual-input CNN technique to identify different stages of sleep using the Sleep-EDF database. Considering data variability and data inefficiency as two of the bottlenecks towards effective model development, a deep transfer learning approach introduced in [14] can transfer information from a large dataset to a small cohort for autonomous sleep staging. However, learning from raw polysomnography signals or time-frequency image representations simultaneously is challenging and not well explored. XSleepNet, a sequence-to-sequence sleep staging model, is proposed in [15] and can learn a combined representation from both raw signals and time-frequency images. The performance of any automatic sleep stage classification can be limited on a specific PSG montage and, hence, shows very poor performance on new data with different demographics. To overcome these challenges, a new deep learning model called RobustSleepNet has been introduced in [16]. It can handle any PSG montage and is capable of accurate sleep stage classification even on unseen data with different demographic characteristics. An end-to-end network model combined with a multi-branch CNN and a residual attention technique has been proposed in [17]. The authors have ensured the end-to-end processing of the multi-channel sleep signals in their proposed scheme and demonstrated that this approach can achieve improved model classification accuracy. A temporal CNN incorporated with a “Conditional Random Field” layer has been proposed in order to achieve improved performance [18]. The authors also added a data augmentation technique to enhance the CNN’s training, which has auxiliary effects. Using single-channel EEG inputs, the authors present a unique attention-based deep learning architecture in another study dubbed AttnSleep to categorize different stages of sleep [19]. The proposed architecture begins with a feature extraction module based on an adaptive feature recalibration (AFR) and a multi-resolution convolutional neural network (MRCNN). Another study [20] suggests to use an automated deep neural network using a multi-model integration approach and multiple input signal channels. A sleep stage classification method has been proposed that involves extracting features from EEG signals using an improved model-based essence feature extraction technique [21]. These features are then used to train a support vector machine (SVM) classifier to classify sleep stages. 

Apart from the CNN-based scheme, an RNN-based model is also developed for sleep stage scoring. RNNs can capture temporal information from sequences of inputs, by employing an internal memory block, and utilize feedback (or loop) connections. Michielli et al. proposed a new method for automatically scoring sleep stages using a single-channel EEG signal [22]. This method uses a cascaded recurrent neural network (RNN) architecture with long short-term memory (LSTM) blocks. A total of 55 time- and frequency-domain features are extracted from the EEG signal and then reduced to select the most important features. The selected features are then used as inputs for the LSTM networks. The cascaded architecture consists of two LSTM RNNs. The first network classifies the five sleep stages (with N1 and rapid eye movement (REM) stages merged) with a 90.8% accuracy rate, while the second network achieves an 83.6% accuracy rate for distinguishing between N1 and REM stages. Mousavi et al. presented a novel approach, SleepEEGNet, for automatically annotating sleep stages using a single-channel EEG signal [23]. This method utilizes a deep CNN to extract time-invariant features and frequency information, as well as a sequence-to-sequence model to capture long-term context dependencies between sleep epochs and scores. In addition, new loss functions have been employed to ensure an equal misclassified error for each sleep stage during network training and, hence, can handle the issue of class imbalance in existing sleep datasets. In [24], Sokolovsky and colleagues developed an automated system for classifying sleep stages using EEG and EOG signals. Their approach differed from other studies by using two EEG channels and one EOG channel, and the input data was in the form of a 2D array with a shape (15,000, 3) consisting of three 150 s signals sampled at 100 Hz. In [25], an LSTM-based classifier was developed to detect sleep stages from a single-lead electrocardiogram (ECG) signal. The model calculated heart rate variability and respiratory signals from the ECG, and 25 features were extracted before training. Urtnasan proposed a deep convolutional recurrent model for automatic sleep stage scoring based on raw single-lead ECG data [26]. This model outperformed several previous studies in this field. Previously, we have presented an end-to-end convolutional RNN that takes data from two channels of a PSG recording as input and classifies sleep stages [27]. The performance of the proposed model has been investigated for different RNN variants. 

## 3. Materials and Methods

**Figure 1:**
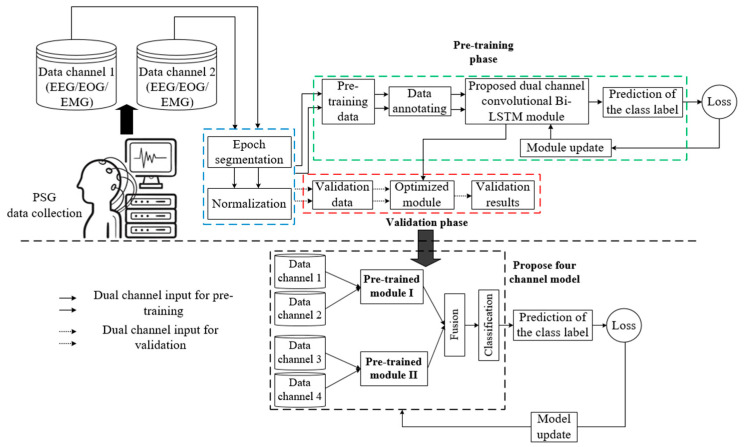
Architecture of the sleep staging using the proposed four-channel convolutional Bi-LSTM network.

This section broadly presents automatic sleep stage detection using the proposed four-channel convolutional Bi-LSTM network with dataset description; data preprocessing, which includes epoch segmentation, data annotation, data normalization, and data splitting; and a detailed demonstration of the network model. Figure 1 depicts the overall architecture of the sleep staging comprising a pre-training dual-channel convolutional Bi-LSTM network module and a post-training four-channel network concatenating two pre-trained dual-channel convolutional Bi-LSTM network modules. However, each essential component of the model depicted above is elaborately demonstrated in the following sections.

### 3.1. Sleep EDF Database Description

This study used two public datasets, Sleep EDF-20 and Sleep EDF-78, which are available in the Physio Bank [31,32]. Sleep EDF-20 contains data files collected from 20 subjects (10 males and 10 females) aged 25–34, while Sleep EDF-78 is an extended version that includes 197 whole-night PSG sleep recordings from 78 healthy Caucasian subjects aged 25–101, which includes EEG, EOG, chin EMG, and event markers, and some also contain respiration and body temperature data. The Sleep EDF dataset comprises two investigations, namely Sleep-Telemetry (ST) and Sleep-Cassette (SC*). ST involved individuals who had difficulty in initiating sleep and consisted of 44 nights, with half of them involving the intake of temazepam and the others involving a placebo. On the other hand, SC involved individuals who did not consume any sleep-related medication and included 153 nights of data. In this study, EOG and EEG signals were sampled at 100 Hz, and submental-EMG signal, oronasal airflow, rectal body temperature, and event markers were sampled at 1 Hz. In both studies, the sleep stage scoring {e.g., wakefulness (W), NREM 1 (N1), NREM 2 (N2), NREM 3 (N3), REM, movement, and unknown} was manually conducted by expert neurologists based on the R&K standards [4].

### 3.2. Epoch Segmentation and Data Annotating

**Figure 2:**
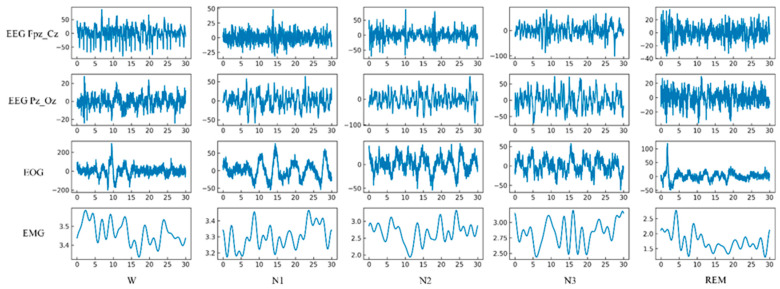
Segmented epochs of five classes for the four channels (EEG Fpz-Cz, EEG Pz-Oz, EOG, and EMG) of the PSG signal.

Two well-experienced neurologists independently annotated sleep scores taking 30 s epochs in each channel of the PSG recording based on the R&K standards. The resulting Kappa score was 0.85, indicating good agreement between the two examiners. A third neurologist confirmed the final scoring. The data was also epoched into 20 s and 10 s intervals in several clinical practices to investigate how a shorter epoch length would affect sleep stage classification. In this study, five sleep stage annotations, including W, S1, S2, S3, and REM stages, are considered. N1 and N2 are denoted as S1 and S2, respectively. Following the data annotation methods in other existing studies, N3 and N4 stages were considered S3 collectively, and the movement and unknown categories were excluded. No artifact rejection was applied to avoid signal distortion. The intuition behind the five-stage characterization is as follows: During the initial stage of sleep, there is a significant presence of alpha and beta waves, frequent eye movements, and muscle activity. In the next stage, more than 50% of alpha waves disappear, eye movements decrease, and vertex waves appear. The presence of sleep spindles and K-complexes are observed in the third stage. The fourth stage is characterized by high amplitude delta waves. The fifth stage, also known as REM sleep, is marked by saw-tooth waves and rapid eye movement. Figure 2 depicts the segmented epochs with their respective annotations for the four channels (EEG Fpz-Cz, EEG Pz-Oz, EOG, and EMG) of the PSG signal.

### 3.3. Data Normalization and Splitting

The training performance of any DL-based model largely depends on data normalization. The objective of applying normalization is to convert data of different channels into the same scale [33]. The data value ranges of every channel from the PSG signal are not similar. Utilizing the raw data samples without normalization can lead to poor generalization of the DL-based model. Among various available data normalization techniques, Min–Max normalization is one of the most popular and overly used data normalization techniques in the literature, and, hence, it has been utilized in this letter too. It is a technique that linearly transforms the variables, and the normalized value of each data point can be computed as follows:

(1) $$x_{i}^{N}=\frac{x-x_{min}}{x_{max}-x_{min}}$$

where $x_{min}$ and $x_{max}$ are the minimum and maximum values of x, respectively, and x is the set of observed values of x. After having the normalized data, we split the entire dataset into the training set and test set as a ratio of 85% and 15%. 

### 3.4. Four-Channel Convolutional Bi-LSTM Network

As depicted in Figure 1, the proposed network model consists of two similar dual-channel convolutional Bi-LSTM network modules, fused or concatenated together to predict the class of sleep stage exploiting the extracted features from four distinct channel signals of PSG recording. The dual channel convolutional is fed by the normalized training samples as part of pre-training. At the early phase of pre-training, the module prediction produces a large loss. In order to reduce the overall loss and improve accuracy, an optimizer is used, which modifies the attributes of the module (e.g., learnable weight). Thus, the module becomes updated after each iteration of the pre-training. Details of implementation are described in Section 4. 

However, the building block of the dual-channel convolutional Bi-LSTM network is “Conv Block”, which consists of two consecutive 1D convolutional layers, a max pooling (Max pool) layer, and a dropout layer, as well as a “Bi-LSTM Block”, which consists of a bidirectional LSTM layer, a max pooling layer, and a dropout layer. Additionally, concatenation layers, a flatten layer, and a dense layer are also used in this proposed architecture.

**Figure 3:**
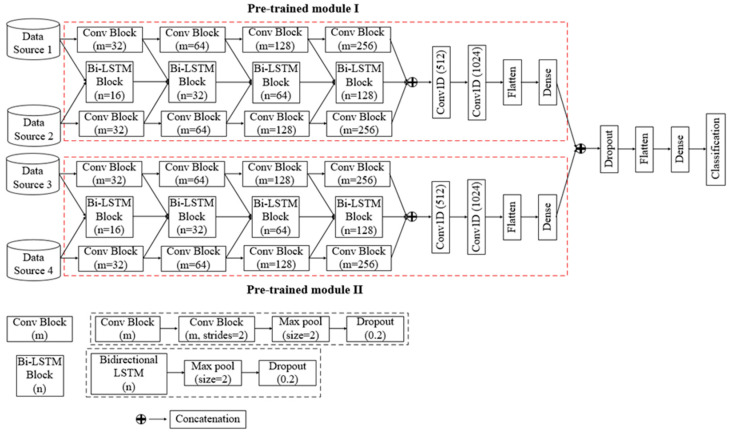
Architecture of the four-channel convolutional Bi-LSTM network.

Figure 3 depicts the architecture of the proposed network. In the dual-channel convolutional Bi-LSTM network module, four consecutive “Conv Block’s” are employed for each data channel to extract spatial features. Later, these extracted spatial features are coupled with the input at every level of the Bi-LSTM network. In the “Conv Block”, the number of filters (m) of the 1D convolutional layer are chosen as 32, 64, 128, and 256 sequentially so that the network does not become computationally expensive and that the features can be coupled to use as input for the Bi-LSTM network. A Max pool layer with a pool size of 2 is used for pooling high contrasting information from the features map, which leads to the down-sampling of the feature map size. In addition, a dropout layer with a dropout rate of 0.2 is also used to overcome the overfitting issue during training. In each 1D convolutional layer, the rectified linear unit activation function is utilized. Now, four Bi-LSTM blocks are employed to extract and learn rich temporal correlated features from dual-channel data. In the “Bi-LSTM Block”, the number of output units of the Bi-LSTM layer are selected as 16, 32, 64, and 128 so that the output can be coupled with the output of “Conv Block”. Additionally, a Max pool layer and a dropout layer with the same dimensions as the “Conv Block” are also inserted in the “Bi-LSTM Block”. However, a Bi-LSTM layer is an extended version of traditional LSTM layer where two LSTMs are employed to extract temporal features in both directions backwards (from future to past) or forward (from past to future) [34]. For a concatenated multi-feature signal $x\left(t\right)$ at time *t*, the corresponding output $h\left(t\right)$ of an LSTM layer can be calculated as follows [35]:

(2) $$f\left(t\right)=\sigma \left(\omega_{f}\cdot \left[h\left(t-1\right),x\left(t\right)\right]+\beta_{f}\right)$$

(3) $$i\left(t\right)=\sigma \left(\omega_{i}\cdot \left[h\left(t-1\right),x\left(t\right)\right]+\beta_{i}\right)$$

(4) $$o\left(t\right)=\sigma \left(\omega_{o}\cdot \left[h\left(t-1\right),x\left(t\right)\right]+\beta_{o}\right)$$

(5) $$c\left(t\right)=f\left(t\right)⊙c\left(t-1\right)+i\left(t\right)⊙tanh\left(\omega_{c}\cdot \left[h\left(t-1\right),x\left(t\right)\right]+\beta_{c}\right)$$

(6) $$h\left(t\right)=o\left(t\right)⊙tanh\left(c\left(t\right)\right)$$

where $f\left(t\right)$, $i\left(t\right)$, and $o\left(t\right)$ denote the forget gate, input gate, and output gate of the LSTM cell, respectively. $c\left(t\right)$ denotes the internal state of an LSTM layer. Now, if $\vec{h\left(t\right)}$ and $\overset{\leftarrow }{h\left(t\right)}$ are the cell state values of two LSTMs produced from the *N* length sequential input and are operated in the backward and forward directions, respectively, the output response of the Bi-LSTM can be derived as follows [34]:

(7) $${\hat{y}}_{t}=concat\left[g\left(\vec{h\left(t\right)}\;\right),\;g\left(\overset{\leftarrow }{h\left(t\right)}\right)\right]$$

where $g\left(.\right)$ denotes corresponding activation function.

## 4. Experimental Results

This section first describes the implementation details of this study along with the evaluation metrics for evaluating the proposed model’s performances. Finally, the overall performances, along with a comparison to other existing network performances, have been presented to investigate the effectiveness of the proposed model and unveil its superiority over other existing networks. 

### 4.1. Implementation Details and Performance Evaluation Metrics 

As described earlier, both the Sleep EDF-20 and Sleep EDF-78 datasets have been utilized in this letter to investigate the performance of the proposed model. At first, the dual-channel two-layer CNN incorporated with the Bi-LSTM network module has been trained by taking combinations of two distinct channels from the Sleep EDF-20 dataset, and, thus, four pre-trained modules for every two distinct channels (e.g., EEG Fpz-Cz + EOG, EEG Fpz-Cz + EMG, EEG Pz-Oz + EOG, and EEG Pz-Oz + EMG) have been prepared for the next step. The EEG Fpz-Cz + EEG Pz-Oz combination has been avoided because they are from the same family (e.g., EEG). After pre-training, every combination of two pre-trained modules (e.g., pre-trained module I and pre-trained module II) is incorporated and retrained again to make the final sleep stage classification. Thus, the overall model training comprises two parts: pre-training of the dual-channel module and retraining the concatenated four-channel convolutional Bi-LSTM network. During retraining, the parameters of the pre-trained dual-channel module were kept unchanged. A similar approach has been utilized to evaluate the performance of the Sleep EDF-78 dataset. However, after having the raw dataset, the data has been prepared following the instruction presented in Section 3.2 and Section 3.3. During the pre-processing of the Sleep EDF-78 dataset, we have excluded “SC4362F0-PSG.edf” and “SC4362FC-Hypnogram.edf” data files because these files were either corrupted or damaged while reading the entire dataset. Next, the proposed model was trained using the training data samples and validated using the test set in parallel. Table 1 presents total number of epochs for each dataset used for training and testing. An Adam optimizer, along with a categorical cross-entropy loss function, was used during the training of the proposed model. Once the training of the model finished, the performance was evaluated using the test data samples. All training and testing programs have been performed in an Anaconda Python 3.7 environment on a system equipped with a 3.80 GHz CPU, 256 GB RAM, and a single Nvidia Quadro RTX 6000 GPU. However, the batch size = 256 and learning rate = 0.001 were kept unchanged during the training and validation. A total of 120 epochs were used to complete the training, in which 80 epochs were used to pre-train the dual channel module, and 40 epochs were used to retrain the overall four-channel network. All the hyper-parameters used to train the proposed model are summarized in Table 2.

In order to compute the performance, four standard statistical indices, also known as evaluation metrics (i.e., accuracy (*ACC*), *F1 score* computed from the positive predictive value (PPV) and sensitivity (SE), and Kappa (*Kp*)) have been adopted in this study. These metrics can be defined as follows [10]:

(8) $$ACC_{i}=\frac{TP_{i}+TN_{i}}{TP_{i}+TN_{i}+FP_{i}+FN_{i}}$$

(9) $$PPV_{i}=\frac{TP_{i}}{TP_{i}+FP_{i}}$$

(10) $$SE_{i}=\frac{TP_{i}}{TP_{i}+FN_{i}}$$

(11) $$F1\;score_{i}=\frac{2PPV_{i}\cdot SE_{i}}{PPV_{i}+SE_{i}}$$

(12) $$Kp_{i}=\frac{ACC_{avg}-P_{e}}{1-P_{e}}$$

where $TN_{i}$ and $FP_{i}$, also known as true negative and false positive, respectively, are the number of other classes that are not classified as the i-th class and the number of other classes that are predicted as the i-th class, respectively. On the other hand, $TP_{i}$ and $FN_{i}$, also known as true positive and false negative, respectively, refer to the number of the i-th class correctly predicted and the number of the i-th class classified into other classes, respectively. In addition, $ACC_{avg}$ represents the average accuracy for the total number of classes, and $P_{e}$ can be termed as the hypothetical probability of agreement by chance. 

### 4.2. Performance Analysis

**Figure 4:**
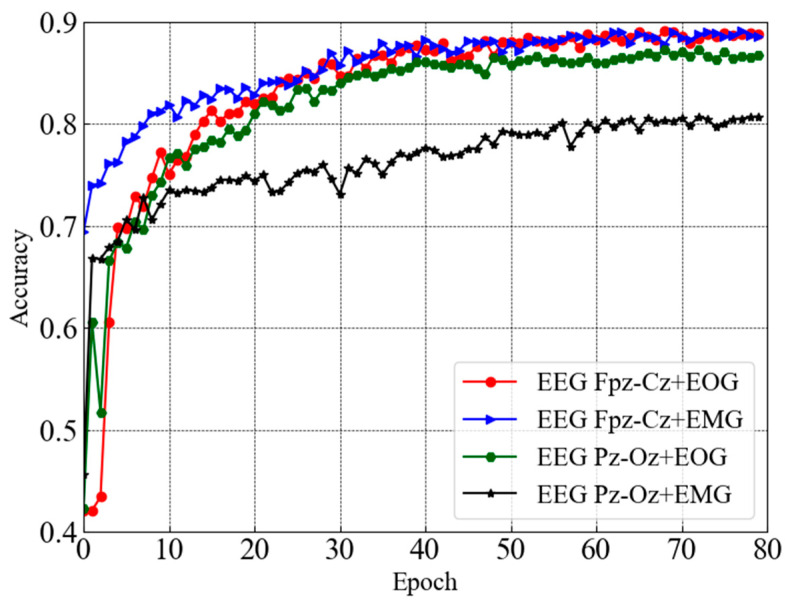
Test accuracy curves for dual-channel modules taking combinations of two distinct channels from Sleep EDF-20 dataset.

This section presents the performance of sleep staging elaborately along with the comparison. Figure 4 depicts test accuracy (e.g., validation accuracy) for dual-channel modules taking combinations of two distinct channels (e.g., EEG Fpz-Cz + EOG, EEG Fpz-Cz + EMG, EEG Pz-Oz + EOG, and EEG Pz-Oz + EMG) from the Sleep EDF-20 dataset. It can be seen that each dual-channel module converged with higher accuracy except one module that takes EEG Pz-Oz + EOG as input after 80 epochs. However, the trends of the accuracy curves also ensure that the learning process is smooth. Convergence with a higher accuracy is observed for the EEG Fpz-Cz + EOG and EEG Fpz-Cz + EMG modules. 

**Figure 5:**
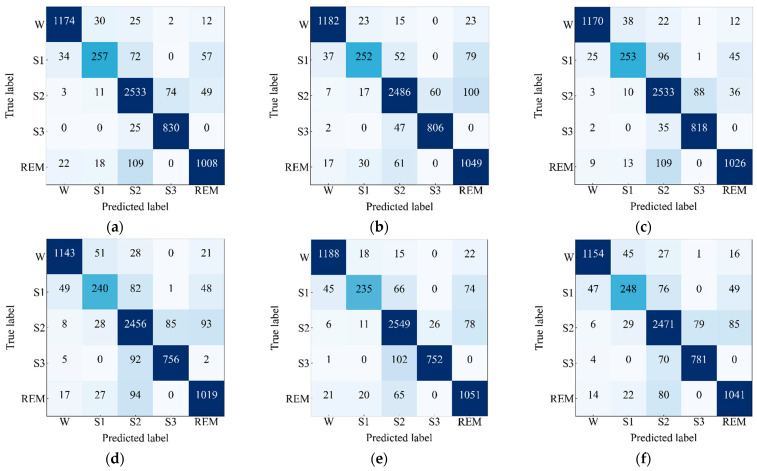
Confusion matrices of the proposed model for (**a**) EEG Fpz-Cz + EOG and EEG Fpz-Cz + EMG, (**b**) EEG Fpz-Cz + EOG and EEG Pz-Oz + EMG, (**c**) EEG Fpz-Cz + EMG and EEG Pz-Oz + EOG, (**d**) EEG Pz-Oz + EOG and EEG Pz-Oz + EMG, (**e**) EEG Fpz-Cz + EOG and EEG Pz-Oz + EOG, and (**f**) EEG Fpz-Cz + EMG and EEG Pz-Oz + EMG applied on Sleep EDF-20 dataset.

Next, confusion matrices have been observed and depicted in Figure 5 to exhibit the model performance on the test set of Sleep EDF-20 dataset. The performance metrics have been computed and are presented in Table 3 by taking values of $TP_{i}$, $FP_{i}$, $TN_{i}$, and $FN_{i}$ from the confusion matrices. The performance metrics for the four-channel convolutional Bi-LSTM network that consists of the EEG Fpz-Cz + EOG and EEG Fpz-Cz + EMG modules as well as the network that consists of the EEG Fpz-Cz + EOG and EEG Pz-Oz + EMG modules are very similar. The highest values of the overall *ACC*, overall *Kp*, and overall *F1 score* are 91.44%, 0.89, and 88.69%, respectively, which is found for the network that includes EEG Fpz-Cz + EOG module and the EEG Fpz-Cz + EMG module. Additionally, the highest *F1 score* for the most skewed class “S1” is 69.83%, which indicates that the network consisting of the EEG Fpz-Cz + EOG module and the EEG Fpz-Cz + EMG module can learn slightly better features for the skewed class. The network that includes the EEG Pz-Oz + EMG module shows poor performance because of its low convergence performance. The network that consists of EEG Fpz-Cz + EMG and EEG Pz-Oz + EMG achieves a lower value of *ACC, Kp*, and *F1 score* (e.g., 91.01%, 0.88, and 87.39%, respectively) compared to others. In addition, the lowest value for of *F1 score* for the most skewed class “S1” is 64.92%, which is achieved for the network consisting of EEG Fpz-Cz + EMG and EEG Pz-Oz + EMG.

**Figure 6:**
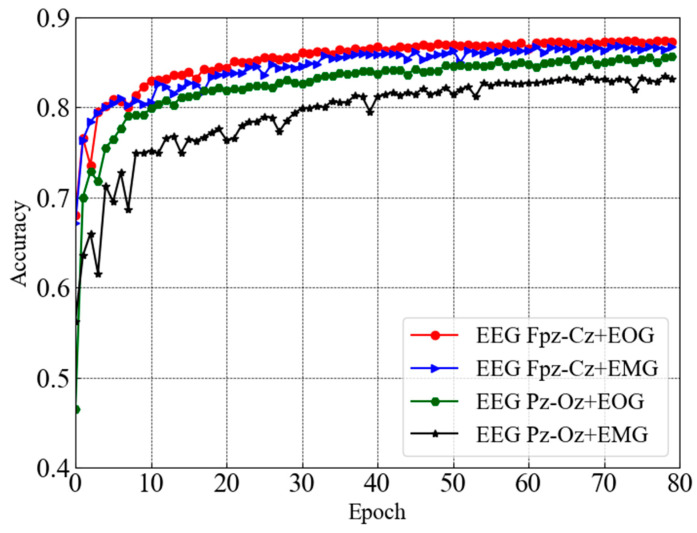
Test accuracy curves for dual-channel modules taking combinations of two distinct channels from Sleep EDF-78 dataset.

Figure 6 depicts the subsequent test accuracy (e.g., validation accuracy) for the dual-channel modules taking combinations of two distinct channels (e.g., EEG Fpz-Cz + EOG, EEG Fpz-Cz + EMG, EEG Pz-Oz + EOG, and EEG Pz-Oz + EMG) from the Sleep EDF-78 dataset. Similar to the above case, the convergence of the EEG Pz-Oz + EOG module is slow compared to the other modules. In the case of the Sleep EDF-78 dataset, the convergence rate of the EEG Fpz-Cz + EMG module is relatively higher than others.

**Figure 7:**
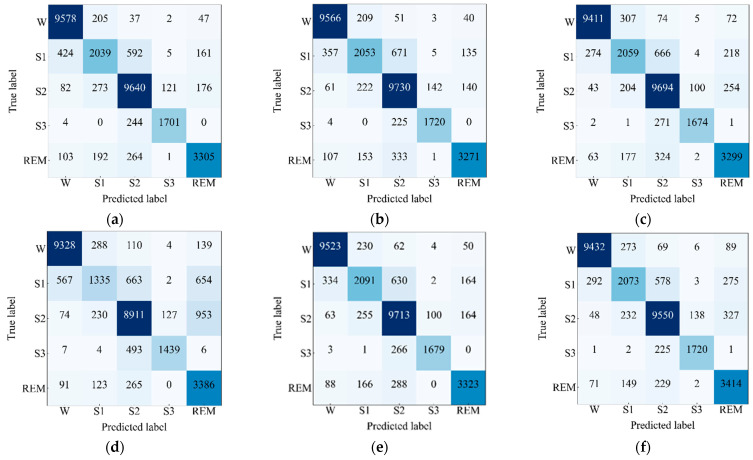
Confusion matrices of the proposed model for (**a**) EEG Fpz-Cz + EOG and EEG Fpz-Cz + EMG, (**b**) EEG Fpz-Cz + EOG and EEG Pz-Oz + EMG, (**c**) EEG Fpz-Cz + EMG and EEG Pz-Oz + EOG, (**d**) EEG Pz-Oz + EOG and EEG Pz-Oz + EMG, (**e**) EEG Fpz-Cz + EOG and EEG Pz-Oz + EOG, and (**f**) EEG Fpz-Cz + EMG and EEG Pz-Oz + EMG applied on Sleep EDF-78 dataset.

At Next, the confusion matrices on the test set of Sleep EDF-78 dataset have been observed and depicted in Figure 7. The performance metrics have been computed and presented in Table 4 taking values of $TP_{i}$, $FP_{i}$, $TN_{i}$ and $FN_{i}$ from the confusion matrices. The highest value of *ACC*, *Kp*, and *F1 score* are 90.21%, 0.86, and 87.02%, respectively, which is found for the network that includes the EEG Fpz-Cz + EMG module and the EEG Pz-Oz + EOG module. Additionally, the highest score of *F1 score* for the most skewed class “S1” is 70.09%, which indicates that the network consisting of the EEG Fpz-Cz + EMG and EEG Pz-Oz + EOG modules has a tendency to capture and learn more enriched features for large data. In this investigation, we have observed a lower performance for the model that includes the EEG Fpz-Cz + EOG and EEG Pz-Oz + EMG modules. Additionally, this network also achieves the lowest *F1 score* (e.g., 51.34%) for the most skewed class, “S1”. However, the difference between the performances is trivial, which validates the efficacy of the proposed model. Moreover, the presence of the EEG Fpz-Cz + EMG module in the network assists the network to show a higher performance and vice versa in the case in which the EEG Pz-Oz + EMG module is present. 

Finally, we have compared the proposed method with other existing works on the Sleep EDF-20 and Sleep EDF-78 datasets. First, the performance achieved on the Sleep EDF-20 dataset has been compared with other existing works [11,12,13,14,15,16,17,20], as shown in Table 5. The table includes results from nine studies, including our proposed network consisting of the Fpz-Cz + EOG module and the Fpz-Cz + EMG module. Table 5 depicts that the proposed network outperformed all other studies in terms of overall performance and class-wise performance for Wake, S2, S3, and REM sleep stages, except for Stage 1, where these values are slightly lower than those reported by Tianqi et al. [16]. Additionally, the performance achieved on the Sleep EDF-78 dataset has also been compared with other existing works [15,16,18,19,23] in Table 6. The table includes the results from six studies, including our proposed network consisting of the EEG Fpz-Cz + EMG module and the EEG Fpz-Cz + EOG module. Compared to other studies, our proposed scheme outperforms in all metrics. More specifically, our proposed scheme can achieve significantly improved results in class S1 compared to others.

## 5. Conclusions

This article proposes a novel approach for automatic sleep stage detection using a multi-channel convolutional bidirectional long short-term memory (Bi-LSTM) network. The aim is to improve the accuracy of sleep stage detection by using data from multiple channels of polysomnography (PSG) recordings, including EEG Fpz-Cz, EEG Pz-Oz, EOG, and EMG. The proposed approach employs a dual-channel convolutional Bi-LSTM network to pre-train the network for every two distinct channels of the PSG recording. The concept of transfer learning is then used to fuse the two dual-channel convolutional Bi-LSTM network modules to detect sleep stages. The study exploits both the Sleep EDF-20 and Sleep EDF-78 datasets, and the proposed model achieves high accuracy, Kappa, and F1 score values for sleep stage classification. Specifically, the network consisting of the EEG Fpz-Cz + EMG module and the EEG Pz-Oz + EOG module exhibits the best performance. The proposed approach is compared with other existing literature, and the results confirm the efficacy of the proposed model. The overall results validate that the proposed scheme can be regarded as an alternative to conventional sleep staging.

## Figures and Tables

**Table 1:** Illustration of the PSG (EEG, EMG, and EOG) data used in this experiment.

Sleep Stage	Symbol	Sleep EDF-20		Sleep EDF-78	
		Training Epochs	Test Epochs	Training Epochs	Test Epochs
Awake	W	7042	1242	55,925	9869
Stage 1	S1	2384	420	18,248	3220
Stage 2	S2	15,129	2670	58,338	10,295
Stage 3 and Stage 4	S3	4848	855	11,042	1949
Rapid Eye	REM	6559	1158	21,901	3865

**Table 2:** Hyper-parameters used to train the proposed model.

Name of the Hyper- Parameters	Value
Training data shape	(3000, 1)
Iteration	80 for pre-trained dual-channel modules and 40 for training the whole architecture
Optimizer	Adam
Batch size	256
Loss function	Categorical cross-entropy
Learning rate	0.001

**Table 3:** Classification performance of the proposed model applied on Sleep EDF-20 dataset.

PSG Channel	Overall Performance			Class-Wise Performance (*F1 Score*) (%)				
	*Acc* *(%)*	*Kp*	*F1 score (%)*	W	S1	S2	S3	REM
EEG Fpz-Cz + EOG and EEG Fpz-Cz + EMG	91.44	0.89	88.09	94.83	69.83	93.23	94.26	88.30
EEG Fpz-Cz + EMG and EEG Pz-Oz + EOG	91.41	0.88	88.01	95.43	68.93	92.69	92.79	90.16
EEG Fpz-Cz + EOG and EEG Pz-Oz + EMG	91.01	0.88	87.39	95.01	67.92	93.26	93.66	87.13
EEG Pz-Oz + EOG and EEG Pz-Oz + EMG	88.49	0.84	84.43	92.74	62.66	90.59	89.09	87.09
EEG Fpz-Cz + EOG and EEG Pz-Oz + EOG	91.01	0.87	87.05	94.88	66.76	93.21	92.10	88.24
EEG Fpz-Cz + EMG and EEG Pz-Oz + EMG	89.75	0.85	85.95	93.51	64.92	91.62	91.02	88.67

**Table 4:** Classification performance of the proposed model applied on Sleep EDF-78 dataset.

PSG Channel	Overall Performance			Class-Wise Performance				
				*F1 Score* (%)				
	*Acc* *(%)*	*Kp*	*F1 Score* *(%)*	W	S1	S2	S3	REM
EEG Fpz-Cz + EOG and EEG Fpz-Cz + EMG	89.94	0.86	86.65	95.48	68.76	91.49	90.02	87.50
EEG Fpz-Cz + EMG and EEG Pz-Oz + EOG	90.21	0.86	87.02	95.83	70.09	91.34	90.06	87.80
EEG Fpz-Cz + EOG and EEG Pz-Oz + EMG	89.51	0.85	86.18	95.73	68.98	90.92	89.66	85.59
EEG Pz-Oz + EOG and EEG Pz-Oz + EMG	83.56	0.77	0.78	93.57	51.34	85.94	81.74	75.22
EEG Fpz-Cz + EOG and EEG Pz-Oz + EOG	90.17	0.86	87.02	95.80	70.12	91.39	89.93	87.84
EEG Fpz-Cz + EMG and EEG Pz-Oz + EMG	89.69	0.85	86.46	95.69	69.68	91.18	90.09	85.67

**Table 5:** Comparison of the classification performance of existing studies and our studies with Sleep EDF-20 dataset.

Paper	PSG Channel	Overall Performance			Class-Wise Performance (*F1 Score*) (%)				
		*Acc* *(%)*	*Kp*	*F1 Score* *(%)*	W	S1	S2	S3	REM
Supratak et al. [11]	EEG Fpz-Cz	82.0	76.9	76	84.7	46.6	85.9	84.8	82.4
Phan et al. [12]	EEG Fpz- + EOG	82.3	0.75	74.7	-	-	-	-	-
Liu et al. [13]	EEG Fpz-Cz	82.72	0.76	75.91	85	41	88	85	80
	EEG Pz-Oz	80.99	0.73	72.69	85	33	87	82	78
Phan et al. [14]	EEG Fpz-Cz + EOG	84.6	0.782	79.0	82.6	50.0	87.8	86.2	88.4
Phan et al. [15]	EEG Fpz-Cz + EOG	83.3	0.762	77.3	-	-	-	-	-
Guillot et al. [16]	EEG Fpz-Cz + EEG Pz-Oz + EOG	-	-	79.1	-	-	-	-	-
Tianqi et al. [17]	EEG Fpz-Cz + EEG Pz-Oz + EOG + EMG	85.8	0.80	81.2	92.3	54.5	87.8	85.4	85.9
Xiaoqing et al. [20]	EEG Fpz-Cz + EEG Pz-Oz + EOG + EMG	83.6	0.77	78.1	86.4	49.8	88.7	84.5	81.6
**Ours**	**EEG Fpz-Cz + EOG** **and** **EEG Fpz-Cz+EMG**	**91.44**	**0.89**	**88.09**	**94.83**	**69.83**	**93.23**	**94.26**	**88.30**

**Table 6:** Comparison of the classification performance of existing studies and our studies with Sleep EDF-78 dataset.

Paper	PSG Channel	Overall Performance			Class-Wise Performance (*F1 Score*) (%)				
		*Acc* *(%)*	*Kp* *(%)*	*F1 Score* *(%)*	W	S1	S2	S3	REM
Phan et al. [15]	EEG Fpz-Cz + EOG	80.6	0.728	76.7	-	-	-	-	-
Guillot et al. [16]	EEG Fpz-Cz + EEG Pz-Oz + EOG	-	-	76.3	-	-	-	-	-
Khalili et al. [18]	EEG Fpz-Cz	82.46	0.76	76.14	92.4	48.1	84.6	73.8	81.6
	EEG Pz-Oz	79.33	0.71	76.4	87.1	53.0	79.8	72.5	74.2
Eldele et al. [19]	EEG Fpz-Cz	81.3	0.74	75.1	92.0	42.0	85.0	82.1	74.2
Mousavi et al. [23]	EEG Fpz-Cz	80.03	0.73	73.55	91.72	44.05	82.49	73.45	76.06
	EEG Pz-Oz	77.56	68.94	70.00	90.26	42.21	79.71	94.83	72.19
**Ours**	**EEG Fpz-Cz + EMG** **and** **EEG Pz-Oz + EOG**	**90.21**	**0.86**	**87.02**	**95.83**	**70.09**	**91.34**	**90.06**	**87.80**

## Data Availability

All the data used in this study are obtained from public datasets. Readers should be able to obtain those data by requesting the dataset sources described in this study.

## References

[1] Wulff K., Gatti S., Wettstein J.G., Foster R.G. (2010). Sleep and circadian rhythm disruption in psychiatric and neurodegenerative disease. Nat. Rev. Neurosci..

[2] Redmond S.J., Heneghan C. (2006). Cardiorespiratory-based sleep staging in subjects with obstructive sleep apnea. IEEE Trans. Biomed. Eng..

[3] Iber C., Ancoli-Israel S., Chesson A., Quan S.F. (2007). The AASM Manual for the Scoring of Sleep and Associated Events.

[4] Rechtschaffen A., Kales A. (1968). A Manual Standardized Terminology, Techniques, and Scoring System for Sleep Stages of Human Subjects.

[5] Faisal F., Nishat M.M., Mahbub M.A., Shawon M.M.I., Alvi M.M.-U.-H. COVID-19 and its impact on school closures: A predictive analysis using machine learning algorithms. Proceedings of the 2021 International Conference on Science & Contemporary Technologies (ICSCT).

[6] Lim B., Zohren S. (2021). Time-series forecasting with deep learning: A survey. Philos. Trans. R. Soc. A.

[7] Esteva A., Robicquet A., Ramsundar B., Kuleshov V., DePristo M., Chou K., Cui C., Corrado G., Thrun S., Dean J. (2019). A guide to deep learning in healthcare. Nat. Med..

[8] Toma T.I., Choi S. (2022). A Parallel Cross Convolutional Recurrent Neural Network for Automatic Imbalanced ECG Arrhythmia Detection with Continuous Wavelet Transform. Sensors.

[9] Cui Z.H., Zheng X.W., Shao X.X., Cui L.Z. (2018). Automatic sleep stage classification based on convolutional neural network and fine-grained segments. Complexity.

[10] Sors A., Bonnet S., Mirek S., Vercueil L., Payen J.F. (2018). A convolutional neural network for sleep stage scoring from raw single-channel EEG. Biomed. Signal Process..

[11] Supratak A., Dong H., Wu C., Guo Y. (2017). DeepSleepNet: A Model for Automatic Sleep Stage Scoring Based on Single-Channel EEG. IEEE Trans. Neural Syst. Rehabil. Eng..

[12] Phan H., Andreotti F., Cooray N., Chen O.Y., De Vos M. (2019). Joint classification and prediction CNN framework for automatic sleep stage classification. IEEE Trans. Biomed. Eng..

[13] Cai Q., Gao Z., An J., Gao S., Grebogi C. (2021). A Graph-Temporal Fused Dual-Input Convolutional Neural Network for Detecting Sleep Stages from EEG Signals. IEEE Trans. Circuits Syst. II Express Briefs.

[14] Phan H., Chen O.Y., Koch P., Lu Z., McLoughlin I., Mertins A. (2021). Towards More Accurate Automatic Sleep Staging via Deep Transfer Learning. IEEE Trans. Biomed. Eng..

[15] Phan H., Chen O.Y., Tran M.C., Koch P., Mertins A., Vos M.D. (2021). XSleepNet. Multi-view sequential model for automatic sleep staging. IEEE Trans. Pattern Anal. Mach. Intell..

[16] Guillot A., Thorey V. (2021). RobustSleepNet. Transfer Learning for Automated Sleep Staging at Scale. IEEE Trans. Neural Syst. Rehabil. Eng..

[17] Tianqi Z., Wei L., Feng Y. (2020). Multi-branch convolutional neural network for automatic sleep stage classification with embedded stage refinement and residual attention channel fusion. Sensors.

[18] Khalili E., Asl B.M. (2021). Automatic sleep stage classification using temporal convolutional neural network and new data augmentation technique from raw single-channel EEG. Comput. Meth. Programs Biomed..

[19] Eldele E., Chen Z., Liu C., Wu M., Kwoh C.K., Li X., Guan C. (2021). An attention-based deep learning approach for sleep stage classification with single-channel EEG. IEEE Trans. Neural Syst. Rehabil. Eng..

[20] Zhang X., Xu M., Li Y., Su M., Xu Z., Wang C., Kang D., Li H., Mu X., Ding X. (2020). Automated multi-model deep neural network for sleep stage scoring with unfiltered clinical data. Sleep Breath..

[21] Shen H., Ran F., Xu M., Guez A., Li A., Guo A. (2020). An Automatic Sleep Stage Classification Algorithm Using Improved Model Based Essence Features. Sensors.

[22] Michielli N., Acharya U.R., Molinari F. (2019). Cascaded LSTM recurrent neural network for automated sleep stage classification using single-channel EEG signals. Comput. Biol. Med..

[23] Mousavi S., Afghah F., Acharya R. (2019). SleepEEGNet: Automated sleep stage scoring with sequence to sequence deep learning approach. PLoS ONE.

[24] Sokolovsky M., Guerrero F., Paisarnsrisomsuk S., Ruiz C., Alvarez S.A. (2020). Deep Learning for Automated Feature Discovery and Classification of Sleep Stages. IEEE/ACM Trans. Comput. Biol. Bioinform..

[25] Wei Y., Qi X., Wang H., Liu Z., Wang G., Yan X. (2019). A Multi-Class Automatic Sleep Staging Method Based on Long Short-Term Memory Network Using Single-Lead Electrocardiogram Signals. IEEE Access.

[26] Urtnasan E., Park J.-U., Joo E.Y., Lee K.-J. (2022). Deep Convolutional Recurrent Model for Automatic Scoring Sleep Stages Based on SingleLead ECG Signal. Diagnostics.

[27] Toma T.I., Choi S. An End-to-End Convolutional Recurrent Neural Network with Multi-Source Data Fusion for Sleep Stage Classification. Proceedings of the 2023 International Conference on Artificial Intelligence in Information and Communication (ICAIIC).

[28] Durrant-Whyte H.F. (1990). Sensor models and multisensor integration. Autonomous Robot Vehicles.

[29] Abdollahpour M., Rezaii T.Y., Farzamnia A., Saad I. (2020). Transfer Learning Convolutional Neural Network for Sleep Stage Classification Using Two-Stage Data Fusion Framework. IEEE Access.

[30] Siami-Namini S., Tavakoli N., Namin A.S. The Performance of LSTM and BiLSTM in Forecasting Time Series. Proceedings of the 2019 IEEE International Conference on Big Data (Big Data).

[31] Kemp B., Zwinderman A.H., Tuk B., Kamphuisen H.A., Oberye J.J. (2000). Analysis of a sleep-dependent neuronal feedback loop: The slow-wave microcontinuity of the EEG. IEEE. Trans. Biomed. Eng..

[32] Goldberger A.L., Amaral L.A.N., Glass L., Hausdorff J.M., Ivanov P.C., Mark R.G., Mietus J.E., Moody G.B., Peng C.-K., Stanley H.E. (2000). Physiobank, physiotoolkit, and physionet: Components of a new research resource for complex physiologic signals. Circulation.

[33] Sola J., Sevilla J. (1997). Importance of input data normalization for the application of neural networks to complex industrial problems. IEEE Trans. Nuclear Sci..

[34] Alam M.M., Rahman M.H., Ahmed M.F., Chowdhury M.Z., Jang Y.M. (2022). Deep learning based optimal energy management for photovoltaic and battery energy storage integrated home micro-grid system. Sci. Rep..

[35] Wang P., Jiang A., Liu X., Shang J., Zhang L. (2018). LSTM-Based EEG Classification in Motor Imagery Tasks. IEEE Trans. Neural Syst. Rehabil. Eng..

